# Impact of Serum Retinol-Binding Protein 4 Levels on Regulation of Remnant-Like Particles Triglyceride in Type 2 Diabetes Mellitus

**DOI:** 10.1155/2013/143515

**Published:** 2013-03-19

**Authors:** Naoto Yamaaki, Kunimasa Yagi, Junji Kobayashi, Atsushi Nohara, Naoko Ito, Akimichi Asano, Kaoru Nakano, Jianhui Liu, Takuya Okamoto, Yukiko Mori, Azusa Ohbatake, Satoko Okazaki, Yoshiyu Takeda, Masakazu Yamagishi

**Affiliations:** ^1^Department of Internal Medicine, Graduate School of Medical Science, Kanazawa University, 13-1 Takaramachi, Kanazawa 920-8640, Japan; ^2^Department of General Medicine, Kanazawa Medical University, 1-1 Daigaku, Uchinada, Kahoku 920-0293, Japan

## Abstract

*Background*. Although retinol-binding protein 4 (RBP4) associates with insulin resistance and remnant-like particles triglyceride (RLP-TG) elevated in the insulin resistant state, few data exist regarding the relationship between RBP4 and RLP-TG. *Subjects and Methods*. The study included 92 Japanese type 2 diabetic mellitus (T2DM) male patients (age 60.5 ± 13.6 years, body mass index (BMI) 24.7 ± 4.1 kg/m^2^, waist circumference (WC) 88.4 ± 10.7 cm, and HbA1c (NGSP) 7.2 ± 1.9%). Patients on medications affecting insulin sensitivity, including fibrates, biguanides, and thiazolidinedione, were excluded. Visceral fat area (VFA) and subcutaneous fat area (SFA) were measured by computed tomography. *Results*. RBP4 levels showed a significant positive correlation with RLP-TG (*r* = 0.2544 and *P* = 0.0056), TG (*r* = 0.1852 and *P* = 0.041), RLP-TG/TG (*r* = 0.23765 and *P* = 0.0241), and age (*r* = −0.2082 and *P* = 0.0219), although there was no significant correlation with VFA, SFA, adiponectin levels, or homeostasis model of assessment insulin resistance (HOMA-R). Multiple regression analysis revealed that RBP4 was an independent determinant of RLP-TG (*P* = 0.0193) but was not a determinant of TG. *Conclusions*. RBP4 correlates positively with serum RLP-TG independent of fat accumulation in T2DM. RBP4 may regulate remnant metabolism independent of glycemic control in T2DM.

## 1. Introduction

Retinol-binding protein 4 (RBP4) was originally identified as a signal molecule, which transfers the intercellular signals for insulin resistance [1]. Previous clinical studies have reported that RBP4 is associated with the extent of systemic insulin resistance and obesity [2, 3]. RBP4 has been shown to significantly correlate with clinical features relating to insulin resistance and metabolic syndrome, including hyperlipidemia and hypertriglyceridemia [4]. Furthermore, recent studies have shown that RBP4 mainly correlates with dyslipidemic profiles in type 2 diabetes, especially in terms of hypertriglyceridemia. 

The retention of remnant lipoproteins is known to frequently occur in the insulin resistant state and in metabolic syndrome. Impaired remnant metabolism is one of the “residual cardiovascular risk factor” for atherosclerosis [5]. Remnant clearance is retarded in insulin resistant type 2 diabetics resulting in cardiovascular damage [6]. Even though the cardiovascular risk associated with metabolic syndrome has been well established, few data exist concerning the relationship between RBP4 and lipoprotein remnants such as remnant-like particles triglyceride (RLP-TG). Therefore, we are investigating the relationship between RBP4 and serum lipoprotein levels, including RLP-TG, in Japanese diabetic patients. 

## 2. Patients and Methods

### 2.1. Patients

This study was approved by the ethical committee of Kanazawa University. Informed consents were obtained from all subjects. All patients were recruited from Kanazawa University Hospital and its satellite hospital. Of 860 outpatients with type 2 diabetes mellitus, a total of 92 Japanese diabetic men (age 60.5 ± 13.6 years) with their agreement of the computed tomography were included in this study. 

Patients on medications affecting the individual insulin sensitivity, including fibrates, biguanides, and thiazolidinedione, were excluded from this study [7, 8]. Patients with other endocrine diseases or significant renal or hepatic disease were also excluded. Obesity was defined as body mass index (BMI) ≥ 25 kg/m^2^, based on the criteria of the Japan Society for the Study of Obesity [9]. Diabetes mellitus was diagnosed according to World Health Organization criteria [10] and/or receiving medications for diabetes mellitus. 

### 2.2. Laboratory Measurements

BMI was calculated as weight (kg) divided by height (m) squared. Waist circumference (WC) at the umbilical level was measured in the exhalation phase of respiration while standing.

Venous blood samples were performed after a 12-hour overnight fast. Total cholesterol (TC) and triglyceride (TG) were determined by enzymatic methods, and high-density lipoprotein cholesterol (HDL-C) levels were measured by a polyanion-polymer/detergent method. RLP isolation was based on the removal of apo A-I-containing particles and most apo B-containing particles, using an immunoseparation technique, which has been shown to leave remnants of both intestinal and hepatic origin in the unbound fraction. Briefly, monoclonal antibodies to apo A-I and specific monoclonal antibodies to apo B, which do not recognize partially hydrolyzed, apo E-enriched lipoprotein remnants, were immobilized on agarose gel. RLP-TG concentrations were measured in the FHS plasma aliquots that had been frozen at −80°C until the time of analysis. Plasma was incubated with the gel for 2 hours, after which the gel, containing the bound (non-RLP) lipoproteins, was precipitated by low-speed centrifugation. Triglyceride concentrations were then measured in the unbound supinates. Blood glucose was measured with the glucose oxidase method and HbA1c by high-pressure liquid chromatography. Plasma adiponectin levels were measured with an enzyme-linked immunosorbent assay kit (Otsuka Pharmaceutical Co., Tokushima, Japan), and serum RBP4 was measured with Human RBP4 Competitive ELISA kit (Adipogen, Seoul, Republic of Korea).

It is well known that RLP-TG has strongly correlated with TG, which represents both overproduction and catabolism of VLDL. This suggests that RLP-TG levels can reflect VLDL overproduction. Therefore, we examined RLP-TG/TG ratio as the focused index for remnant catabolism. Homeostasis model of assessment insulin resistance (HOMA-R) is calculated using the following formula: fasting glucose (mg/dl) × fasting insulin (*μ*U/mL)/405.

### 2.3. Body Fat Distribution

All subjects underwent computed tomography (CT) at the umbilical level to measure cross-sectional visceral fat area (VFA) and abdominal subcutaneous fat area (SFA) using Fat Scan (N2 System Corp, Osaka, Japan) [11]. VFA/SFA ratio was calculated as VFA divided by SFA.

### 2.4. Statistical Analysis

All data are shown as mean ± SD. Continuous variables were compared by ANOVA after being adjusted for age, BMI, and sex. All statistical analyses were conducted with JMP ver. 10.0.2 for Macintosh OSX (SAS institute, NC, USA). A *P* value of less than 0.05 was considered statistically significant.

## 3. Results

Baseline clinical characteristics of the patients are shown in Table 1. Our previous report showed that poorly controlled type 2 diabetic men had more unfavorable lipid profiles than women, thus resulting in decreased lipolysis of plasma TG-rich lipoproteins [12]. Therefore, only type 2 diabetic men were enrolled in this study.

**Figure 1:**
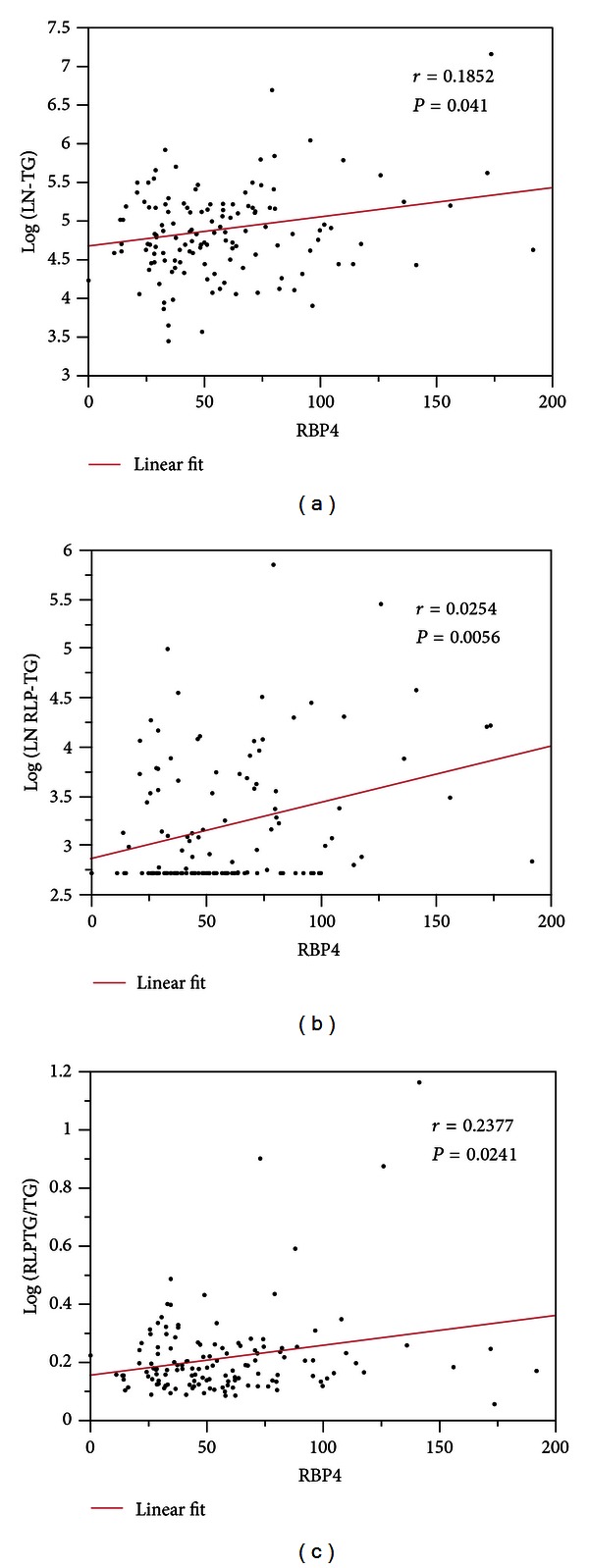
Relationship between serum RBP4 levels and logarithm transformed triglyceride (TG) levels (a), logarithm transformed RLP-TG levels (b), and RLP-TG/TG ratio (c).

Relationships between RBP4 and other clinical parameters were shown in Table 2. RBP4 showed significant positive correlation with TG (*r* = 0.1852 and *P* = 0.041) (Figure 1(a)), RLP-TG (*r* = 0.2544 and *P* = 0.0056) (Figure 1(b)), WC (*r* = 0.1919 and *P* = 0.0336), and negatively correlated with age (*r* = −0.2082 and *P* = 0.0219). RBP4 also showed good correlation with RLP-TG/TG (*r* = 0.2376 and *P* = 0.0241) (Figure 1(c)). Importantly, there was no significant correlation between RBP4 and HOMA-R. 

RLP-TG showed positive correlation with TC (*r* = 0.2894 and *P* = 0.0017), TG (*r* = 0.6732 and *P* < 0.0001), HbA1c (*r* = 0.3506 and *P* = 0.0011), HOMA-R (*r* = 0.3527 and *P* = 0.0054), RBP4 and negative correlation with age (*r* = −0.3377 and *P* = 0.0003), and HDL-C (*r* = 0.3682 and *P* < 0.0001). 

TG showed positive correlation with TC (*r* = 0.4297 and *P* < 0.0001), RLP-TG, HbA1c (*r* = 0.3054 and *P* = 0.0042), negative relationships with age (*r* = −0.2810 and *P* = 0.0023), and HDL-C (*r* = −0.3663 and *P* < 0.0001). Unlike RLP-TG, TG did not have a significant correlation with HOMA-R.

To investigate which factors independently determined RLP-TG and TG, we performed multiple regression analysis. Multiple regression analysis with RLP-TG (log) (Table 3(a)) as an objective variable revealed that RBP4 and age had independent relationships with RLP-TG. Multiple regression analysis with TG as an objective variable showed that VFA and age had independent relationships with TG, but RBP4 had no independent relationships with TG (Table 3(b)).

## 4. Discussion

In the present study, we showed for the first time that serum RBP4 levels correlated with RLP-TG. Univariate analyses showed that RBP4 was significantly associated with RLP-TG and TG. There was no correlation of RBP4 with most of the insulin-resistance-associated factors (adiponectin, VFA, BMI, and HOMA-R) except for a weak correlation with WC, even though most of these factors were significantly correlated with RLP-TG. Furthermore, multiple regression analysis revealed that RBP4 had an independent association with RLP-TG, although RBP4 did not have an independent association with TG levels. These results suggested that RBP4 could regulate TG metabolism especially at the remnant lipoprotein level. 

RBP4 was identified as a signal transferring protein for insulin resistance. Therefore, we focused mainly on its relationship with insulin-resistant states in this study, although some reports have shown that RBP4 could play a more important role in lipid metabolism than in insulin resistance [13]. RBP4 correlates with serum TG levels in type 2 diabetic patients independent from liver fat content [14, 15]. Furthermore, some reports have shown that RBP4 correlates with hypertriglyceridemia independent of insulin resistance [4, 13, 16]. In this study, serum RBP4 levels significantly correlated with parameters relating to RLP-TG and TG. Among the generally accepted markers for insulin resistance, no significant relationships were observed between RBP4 and BMI, VFA, HOMA-R, or adiponectin. WC showed a relatively weak relationship with RBP4. The correlation with WC is indirect and is usually considered as a surrogate marker reflecting the extent of insulin resistance. Actually, the appropriate cutoff value of WC to detect metabolic syndrome is still under discussion [17]. However, it is still unclear why RBP4 correlated only with WC instead of VFA which is closely related to insulin resistance. Imbalance of fat distribution in the study group may explain this dissociation. Even under these conditions, RBP4 can regulate TG metabolism especially at the remnant level, but visceral fat accumulation and factors associated with insulin resistance may not be the main pathway for RBP4 to regulate TG metabolism. 

Two possible mechanisms explaining the increased level of RLP-TG could be presented, one is the decreased metabolism of TG caused by decreased LPL activity induced by insulin resistance [18] and another is the overproduction of TG. Decreased LPL activity accounts for a great deal of the elevation of serum TG in insulin-deficient diabetes patients [19, 20]. As the RLP-TG to TG ratio has been reported to correlate with LPL activity [21], the result of a significant and positive relationship between RBP4 and the RLP-TG to TG ratio in this study suggests that decreased LPL activity would have contributed much to the elevation of RLP-TG. In addition, RBP4 carries ligands of retinoid X receptor (RXR), and RXR modulates the expression of several genes including apoC-III, which is an inhibitor of the lipoprotein lipase [22, 23]. Based on the present findings and previous reports, RBP4 is thought to increase RLP-TG through the activation of RXR, inducing the overexpression of apoC-III and decreasing LPL activity in type 2 diabetic patients.

This study has several limitations. First, we did not measured LPL activity. As the RLP-TG to TG ratio has been reported to correlate with LPL activity [21], we used a surrogate marker of RLP-TG to TG ratios for LPL activity. Although direct measurement would be ideal, our current results seemed to justify using a surrogate marker to represent LPL activity. Second, some patients were treated with insulin or sulphonylureas, which might affect RBP4 metabolism. However, these medicines were discontinued the day prior to the examination, so the effect on remnant metabolism would be minimized. 

In conclusion, RBP4 correlates positively with serum RLP-TG independent of markers reflecting insulin resistance including visceral fat accumulation in patients with type 2 diabetes mellitus. These results suggest that RBP4 may have a negative effect on remnant metabolism independent of visceral fat accumulation, glycemic control, or factors associated with insulin resistance.

## Figures and Tables

**Table 1:** Clinical characteristics of the study subjects.

Age (years old)	60.5 ± 13.6
Body mass index (kg/m^2^)	24.7 ± 4.1
Fasting blood glucose	130.5 ± 33.5
HbA1c (NGSP) (%)	7.2 ± 1.9
Total cholesterol (mg/dL)	203 ± 43
Triglyceride (mg/dL)	165 ± 155
RLP-triglyceride (mg/dL)	34.4 ± 45.4
HDL-cholesterol (mg/dL)	51 ± 16
LDL-cholesterol (mg/dL)	118 ± 30
RBP4 (*μ*g/mL)	60.4 ± 35.0
Adiponectin (ng/mL)	6.84 ± 7.09
HOMA-R	2.88 ± 2.92
Waist circumference (cm)	88.4 ± 10.7
Visceral fat area (cm^2^)	110.6 ± 53.5
Subcutaneous fat area (cm^2^)	138.4 ± 75.2
Treatments for diabetic subjects	
Sulphonylureas	16
Glinides	12
Alpha glucosidase inhibitors	17
Insulin injection	10

All data were indicated as mean ± SD.

**Table 2:** Pearson correlation coefficients in the 92 patients with type 2 diabetes mellitus.

	RBP4	TG	RLP-TG
RBP4	—	0.1852*	0.2544^#^
Triglyceride (TG)	0.1852*	—	0.6732^$^
Remnant-like particle TG (RLP-TG)	0.2544^#^	0.6732^$^	—

Age	−0.2082*	−0.2810^#^	−0.3337^†^
BMI	0.1433	0.0290	0.0824
Total cholesterol (TC)	0.1729	0.4297^$^	0.2894^#^
HDL-C	0.0179	−0.3663^$^	−0.3682^$^
LDL-C	0.1047	0.1531	0.0292
Fasting plasma glucose	0.1214	0.1468	0.1794
HbA1c	0.1268	0.3054^#^	0.3506^#^
Visceral fat area (VFA)	0.1386	0.1295	0.1537
Subcutaneous fat area (SFA)	0.1559	0.0225	0.0460
Waist circumference (WC)	0.1919*	0.0427	0.0976
Adiponectin	0.0842	0.0857	0.1114
HOMA-R	0.0923	0.1670	0.3527^#^

TG and RLP-TG were logarithm transformed. *P < 0.05; ^#^ *P* ≤ 0.01; ^†^ *P* ≤ 0.001; ^$^ *P* ≤ 0.0001.

**Table:** (a)

Factors	Standard error	*t*	*p* (prob > *t*)
Age	0.005372	−3.47	0.0008*
RBP4	0.001999	2.39	0.0193*
VFA	0.001597	1.87	0.0649
Adiponectin	0.010075	−1.59	0.1154
BMI	0.034605	−0.36	0.7208
SFA	0.001799	−0.23	0.8218

**Table:** (b)

Factors	Standard error	*t*	*p* (prob > *t*)
Age	0.004836	−3.59	0.0006*
VFA	0.001434	2.40	0.0189*
BMI	0.031260	−1.64	0.1044
RBP4	0.001792	1.46	0.1485
Adiponectin	0.009033	−1.39	0.1687
SFA	0.001654	−0.19	0.8494
